# The lung-muscle metabolic axis: a proposed mechanism for post-resection systemic frailty

**DOI:** 10.1007/s00595-026-03336-7

**Published:** 2026-06-04

**Authors:** Ichiro Yoshino, Hisashi Saji, Hidemi Suzuki

**Affiliations:** 1 Department of Thoracic Surgery, International University of Health and Welfare Narita Hospital, 867 Hatakeda, Narita, 286-8520 Japan; 2 Department of General Thoracic Surgery, Chiba University Graduate School of Medicine, Chiba, Japan; 3 Department of Thoracic Surgery, St. Marianna University School of Medicine, Kawasaki, Japan

**Keywords:** Non-small cell lung cancer, Pulmonary hypertension, Lung resection surgical metabolism, Sarcopenia, Myokine

## Abstract

Long-term non-cancer mortality following lung cancer surgery remains an under-recognized challenge in clinical practice. This review proposes a novel “pulmonary hypertensive phenotype” hypothesis, suggesting that the permanent reduction of the pulmonary vascular bed after anatomical resection acts as the primary upstream driver of systemic vulnerability. While the ventilatory capacity may partially recover through enlargement of the airspace and compensatory remodeling, restoration of the pulmonary vascular bed is often incomplete. This deficit leads to a sustained right ventricular afterload and chronic endothelial stress. We integrated clinical and experimental evidence to suggest that this cardiopulmonary strain triggers systemic catabolic signaling, potentially mediated by factors such as GDF-15, which accelerates skeletal muscle proteolysis and attrition. This metabolic “phase-shift” can push patients beyond a critical threshold of vulnerability, increasing the risk of sarcopenia and non-cancer death. This could be particularly evident after extensive resections, such as pneumonectomy and lobectomy, compared with sublobar-sparing approaches. Ultimately, surgical invasiveness should be re-evaluated not only by lung volume loss but also by the preservation of the pulmonary vascular bed necessary to maintain systemic homeostasis. This framework provides a biologically plausible pathway linking the extent of resection to long-term systemic resilience and should be regarded as a conceptual integrative model that requires clinical validation.

## Introduction

The history of lung cancer surgery involves balancing oncologic radicality with physiological preservation. The surgical management of lung cancer has progressively evolved toward the optimization of resection. Historically, pneumonectomy was the standard approach for centrally located tumors [1], followed by the establishment of lobectomy with mediastinal dissection as the gold standard [2]. More recently, accumulating evidence from randomized studies, including JCOG0802/WJOG4607L [3] and CALGB 140,503 [4], has supported the oncologic feasibility of segmentectomy in selected early-stage non–small cell lung cancer, and the former indicates a trade-off between cancer curability and the incidence of death from other diseases.

This trajectory reflects a fundamental principle: the lung is a vital organ whose functional reserve, particularly the vascular component, cannot be easily replaced. Unlike the liver or kidney, compensatory mechanisms in the lung are strictly limited by the structural and vascular constraints of pulmonary circulation. While spirometric indices (e.g., FEV₁) are standard for evaluating functional recovery, they primarily reflect aerodynamics and often mask persistent stress on pulmonary circulation. Recent evidence indicates that even when spirometric data normalize post-resection, the reduction in the pulmonary vascular bed creates a sustained hemodynamic load on the right ventricle [5, 6].

Does this reduced pulmonary vascular capacity produce a chronic “hemodynamic phenotype” with systemic metabolic consequences In the context of surgical metabolism, where surgical trauma and subsequent healing demand continuous substrate mobilization, a persistent cardiopulmonary strain may force a long-term redistribution of systemic energy. We propose the existence of a “lung-muscle metabolic axis,” where a post-resection pulmonary hypertensive phenotype triggers interorgan signaling networks, potentially involving endocrine factors like growth differentiation factor-15 (GDF-15), that drive progressive skeletal muscle attrition and systemic frailty.

In this review, we integrate pulmonary vascular physiology, compensatory lung remodeling, and surgical metabolism to explore how the extent of parenchymal resection reshapes the long-term systemic outcomes.

### Compensatory response and its systemic consequences after lung resection

**Fig. 1:**
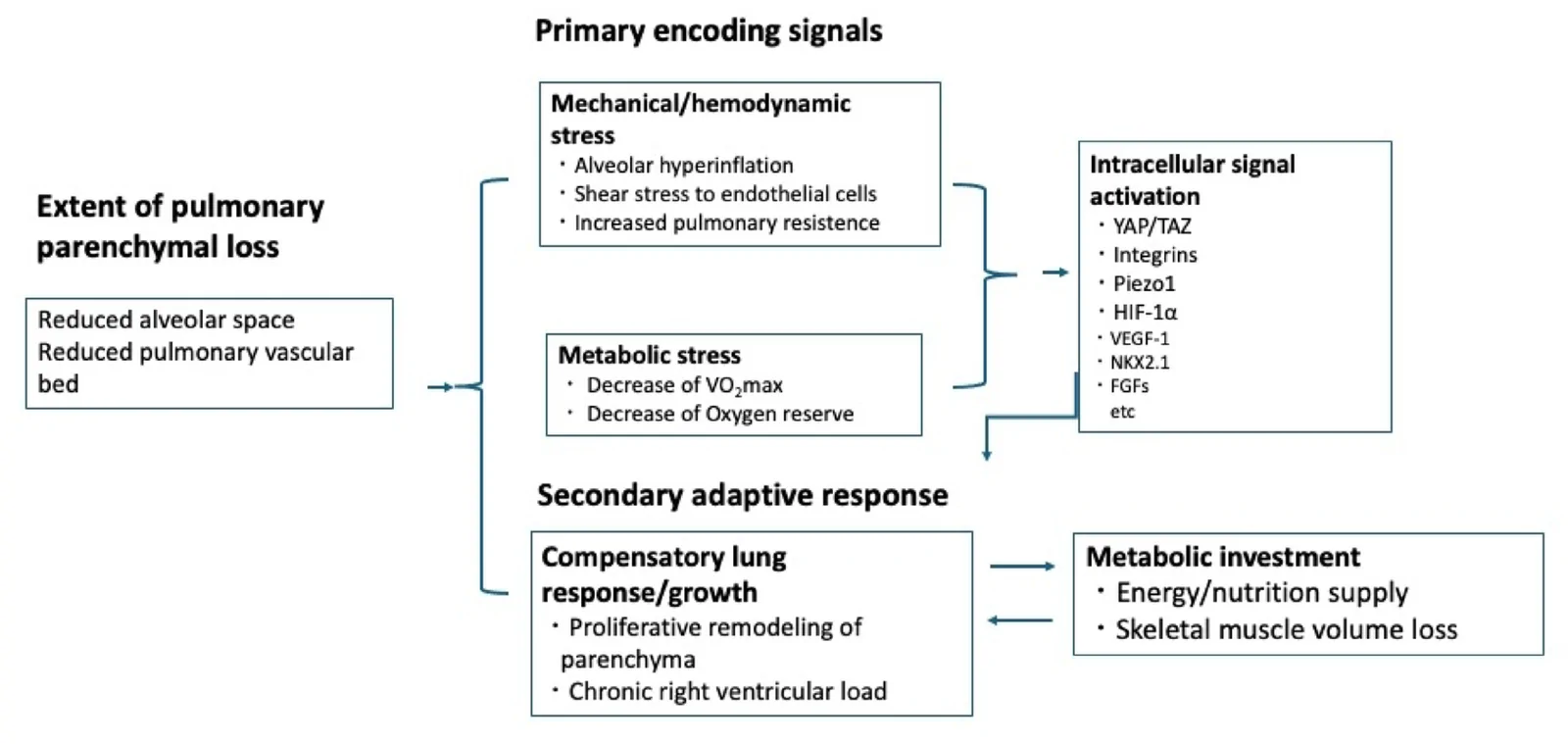
Encoding of lung parenchymal loss into systemic metabolic and endocrine signals. A schematic representation of how the extent of pulmonary parenchymal resection is translated into systemic metabolic and endocrine responses. Lung parenchymal loss reduces the alveolar surface area and pulmonary vascular bed capacity, generating mechanical and hemodynamic stress within the remnant lung. The primary encoding signals include alveolar hyperinflation, endothelial shear stress, and increased pulmonary vascular resistance, leading to reduced oxygen reserve and diminished maximal oxygen uptake (VO₂max). These changes activate the intracellular adaptive pathways, such as YAP/TAZ, integrins, Piezo1, HIF-1α, VEGF, NKX2.1, and FGFs, triggering compensatory lung remodeling and vascular adaptation. However, such remodeling requires metabolic investment, including the diversion of energy and substrates from systemic reserves. Chronic right ventricular load and sustained cardiopulmonary stress further amplify the whole-body metabolic demand. Thus, lung parenchymal loss functions as a quantitative biological signal linking local structural adaptation to the systemic metabolic burden

Anatomical lung resection inevitably reduces the alveolar surface area and pulmonary vascular bed capacity. Postoperative spirometric recovery is often observed according to the extent of the resected parenchyma [5, 7], and structural and hemodynamic adaptation of the remnant lung may occur concurrently. Radiologic analyses of clinical cases have shown increases in remnant lung volume and estimated lung weight after lobectomy and segmentectomy [8, 9], suggesting structural adaptation beyond simple hyperinflation. Experimental studies have consistently demonstrated that compensatory lung remodeling follows parenchymal resection in a magnitude-dependent manner and proliferative remodeling involving epithelial, endothelial, and mesenchymal components [10, 11], mediated in part by mechanotransduction pathways, including YAP/TAZ signaling and hypoxia-responsive mechanisms [12, 13] (Fig. 1).

The presence of pulmonary hypertension in preoperative evaluation is a pathological condition that significantly affects the postoperative course [14], it is also well known that lung resection itself can increase pulmonary vascular resistance because the reduction of the pulmonary vascular bed alters the pressure–flow relationship of the pulmonary circulation. An increased blood flow per unit of lung tissue and augmented endothelial shear stress have been documented after major pulmonary resection [15]. Clinical studies evaluating pulmonary artery wave reflection and right ventricular performance have indicated that right ventricular afterload may increase following lung resection [16,17]. Moreover, assessment using the pulmonary artery–to–aorta (PA/A) ratio suggests that postoperative pulmonary artery enlargement is more pronounced after lobectomy than after segmentectomy and may be associated with long-term survival differences [18, 19].

Importantly, spirometric indices primarily reflect airflow mechanics and do not directly quantify the pulmonary vascular reserve or right ventricular workload. Consequently, the recovery of FEV₁ or vital capacity does not necessarily imply the normalization of cardiopulmonary coupling. The physiological principles of pulmonary vascular mechanics indicate that reduced vascular bed capacity may limit the recruitable reserve during exertion, potentially increasing the relative cardiopulmonary effort even in the presence of preserved resting spirometry [20, 21].

Collectively, these findings support the possibility that lung resection induces a chronic pulmonary vascular phenotype characterized by a reduced vascular reserve and sustained right ventricular load. While the degree of hemodynamic alteration likely varies according to the resection extent and baseline reserve, available data are consistent with a dose-dependent relationship between parenchymal loss and cardiopulmonary stress. Furthermore, such stress on the pulmonary vasculature can lead to tissue remodeling, further increasing the stress and creating a vicious cycle.

### Pulmonary vascular stress as a systemic endocrine signal: linking right ventricular load to skeletal muscle catabolism

Anatomical lung resection inevitably reduces the pulmonary vascular beds. Although compensatory alveolar expansion and structural remodeling partially restore lung volume recovery, the vascular reserve appears incomplete as mentioned previously. Consequently, the pulmonary blood flow per unit of lung tissue increases, and the pulmonary vascular resistance may rise, particularly during exertion. Even when resting spirometric values recover, subtle exercise-induced pulmonary hypertension and right ventricular (RV) load have been documented after lobectomy compared with sublobar resection [22, 23]. Recently, Okada et al. showed through echocardiographic studies that the greater the amount of lung resection, the greater the right heart load during exercise [24].

Chronic RV afterload is not merely a hemodynamic adaptation confined to the cardiopulmonary axis. Sustained pulmonary vascular stress induces endothelial shear stress and mechanotransduction signaling [25], leading to the activation of stress-responsive molecular pathways [26]. In pulmonary hypertension (PH), endothelial dysfunction is associated with the increased production of circulating mediators that extend beyond the pulmonary circulation.

Among these, growth differentiation factor-15 (GDF-15), a member of the transforming growth factor-β superfamily, has emerged as a biomarker and potential effector of cardiopulmonary stress [27]. In patients with pulmonary hypertension, circulating GDF-15 levels correlate with disease severity and adverse outcomes, and pulmonary vascular endothelial cells are considered an important source under conditions of chronic hemodynamic strain [28]. Experimental data further suggest that GDF-15 may exert systemic metabolic effects, including appetite modulation, mitochondrial stress signaling, and promotion of skeletal muscle catabolism [29]. Recently, Semmelmann et al. demonstrated that an increase in serum GDF-15 immediately after surgery is significantly related to pulmonary complications [30]. These findings suggest that pulmonary vascular stress after lung resection, particularly when accompanied by a subclinical pulmonary hypertension (PH) phenotype, may generate endocrine signals that affect peripheral tissues. Although the significance of GDF-15 after lung resection remains an open question, the biological overlap between postresection pulmonary vascular remodeling and established PH pathways suggests the possibility of a common endothelial-muscle signaling axis.

**Fig. 2:**
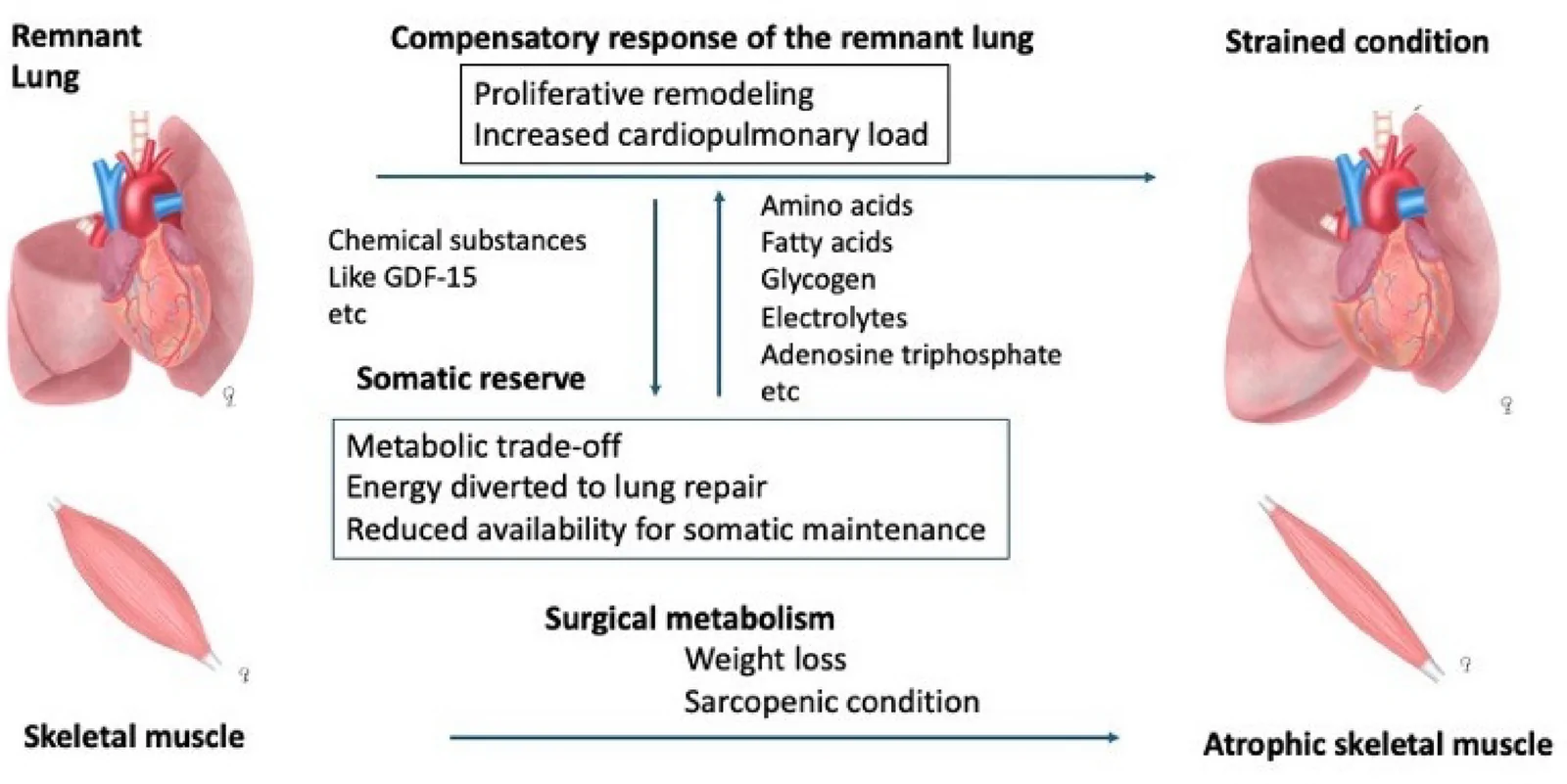
Compensatory response of the remnant lung and systemic cost under surgical metabolism. Conceptual model illustrating the interaction between local lung adaptation and whole-body metabolic allocation following pulmonary resection. Proliferative remodeling of the remnant lung and increased cardiopulmonary workload require substantial energy resources. Within the context of surgical metabolism, characterized by catabolic signaling and substrate mobilization, energy and nutrients are preferentially diverted toward organ repair and cardiopulmonary adaptation. This diversion may reduce the availability of somatic maintenance, contributing to skeletal muscle atrophy and sarcopenic progression. The skeletal muscle functions as a major metabolic reservoir, supplying amino acids, fatty acids, glycogen, electrolytes, and ATP to sustain adaptation. Circulating mediators, including stress-responsive cytokines and factors such as GDF-15, may participate in interorgan communication, linking pulmonary vascular stress to systemic muscle metabolism. The model highlights the energetic competition between the compensatory lung response and the preservation of skeletal muscle mass

Within the broader context of surgical metabolism, such sustained cardiopulmonary adaptation may have systemic implications. Surgical trauma activates neuroendocrine and inflammatory pathways that promote catabolic substrate mobilization [31, 32]. When combined with persistent cardiopulmonary demand and subsequent lung remodeling, energy allocation may be redistributed toward organ-level adaptation. Skeletal muscle, functioning as a principal metabolic reservoir, provides amino acids and energy substrates under catabolic conditions (Fig. 2). Isaka et al. demonstrated that the long-term reduction in psoas muscle mass was significantly greater after lobectomy than after segmentectomy, even among recurrence-free patients, and that the magnitude of muscle loss correlated with the extent of pulmonary resection [33]. Similarly, Takamori et al. reported that the postoperative skeletal muscle loss was independently associated with the inflammatory and nutritional status and a poorer survival after lung cancer surgery [34]. Importantly, these differences were not fully explained by operative time, perioperative complications, or conventional spirometric recovery.[

These observations do not establish a direct causality between pulmonary vascular alterations and skeletal muscle atrophy. However, they are consistent with an integrated model in which cardiopulmonary stress, metabolic investment in lung remodeling, and systemic catabolic signaling interact to influence the long-term muscle integrity and physiological resilience.

### Skeletal muscle as an integrative metabolic organ: energetic competition and phase shift after lung resection

Skeletal muscle is the largest metabolically active organ in the body and functions not only as a locomotive structure but also as a dynamic endocrine and metabolic reservoir [35]. The acute catabolic response in skeletal muscle has been investigated for decades in various conditions; that is, muscle proteolysis supplies amino acids for gluconeogenesis, acute-phase protein synthesis, and tissue repair [36, 37]. The progression of the postoperative sarcopenic state and its impact on prognosis have been demonstrated in several types of surgeries [38–41]. However, lung resection introduces a distinctive and sustained physiological perturbation: permanent loss of the pulmonary vascular reserve combined with ongoing compensatory remodeling. Structural regeneration of the remnant lung, driven by mechanotransduction, angiogenic signaling, and proliferative pathways, requires metabolic investment in the form of energy, substrates, and oxygen delivery. When superimposed on a persistent cardiopulmonary load, this adaptive process may impose a chronic energetic demand.

In this context, skeletal muscle may serve as a systemic buffer. Energetic substrates, such as amino acids, fatty acids, glycogen, and high-energy phosphates, are mobilized to support both cardiopulmonary work and tissue remodeling. If pulmonary vascular stress simultaneously activates endocrine signals that promote muscle catabolism, the combined effect may shift the whole-body metabolic balance toward somatic reserve depletion.

**Fig. 3:**
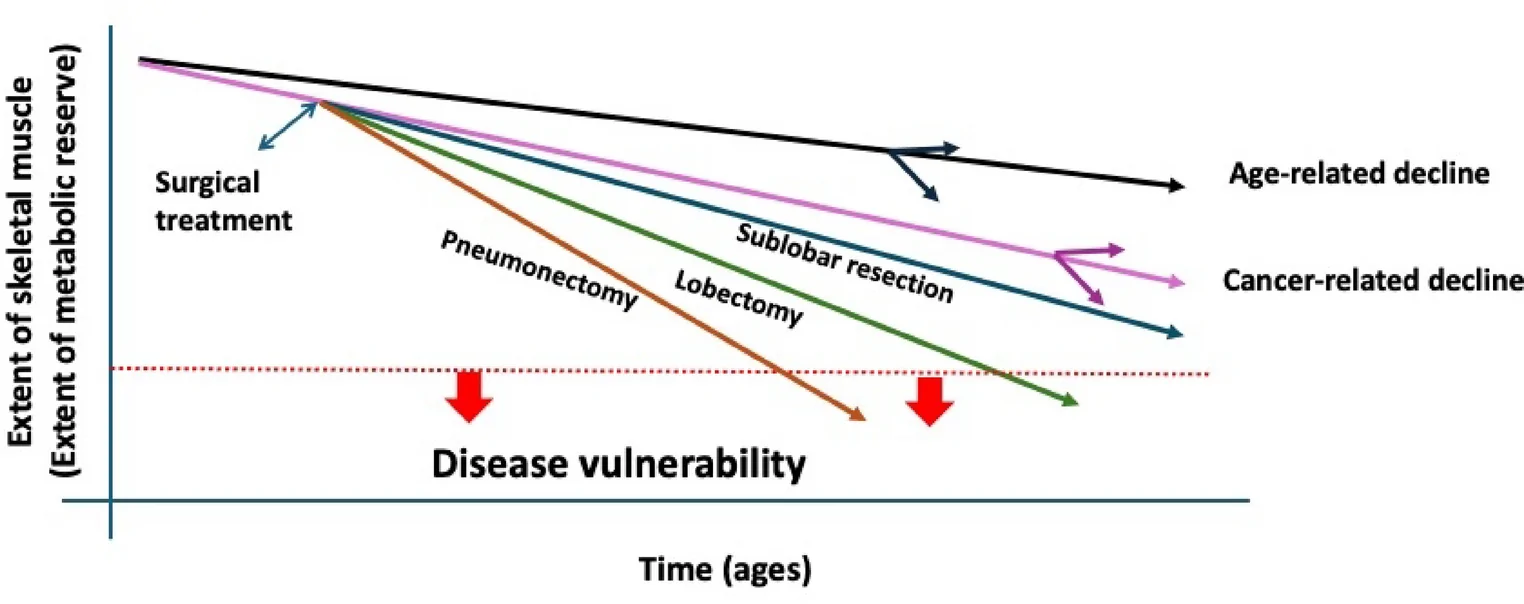
Trajectory of skeletal muscle reserve and disease vulnerability after lung resection. Hypothetical longitudinal model of skeletal muscle mass (as a proxy for systemic metabolic reserve) over time. Normal aging is associated with a gradual decline in muscle mass. Cancer-related systemic stress accelerates this process. Surgical intervention introduces an additional decline, the magnitude of which may vary according to the extent of lung parenchymal resection and the subsequent compensatory metabolic demands. When the skeletal muscle mass falls below a critical threshold, disease vulnerability increases, predisposing patients to non-cancer-related morbidity and mortality. Differences in compensatory burden, pulmonary vascular stress, and physical activity levels may modulate the slope of decline following surgery. This hypothetical framework was originally presented by Williams et al. (ref. number 41. J Clin Oncol. 2021;39:2068-78) regarding cancer patients in general, then illustrated with the same concept in this paper. This figure integrates lung parenchymal preservation, systemic metabolic resilience, and long-term vulnerabilities into a unified model

This framework is consistent with the concept of a “phase shift” in older adult cancer patients (Fig. 3) [42]. Without surgery, skeletal muscle mass gradually decreases due to aging and cancer-related factors, such as decreased activity due to pain, cachexia, appetite loss, and calorie consumption by tumor cells. However, after substantial parenchymal loss due to surgery, the muscle decline slope may accelerate, particularly when cardiopulmonary load and endothelial signaling persist.

Skeletal muscle mass generally declines gradually with increasing age. In patients who have undergone lung resection, significant loss of lung parenchyma may accelerate the rate of muscle mass loss, especially when cardiorespiratory stress and endothelial signaling persist. Importantly, subsequent reductions in physical activity may further exacerbate this trajectory and contribute to the clinical manifestations of respiratory or cardiac sarcopenia [43, 44]. Furthermore, the progression of sarcopenia beyond a certain point may lead to disease vulnerability.

In this integrated model, skeletal muscle loss is not simply a consequence of surgery but an emergent property of chronic cardiopulmonarymetabolic adaptation. Lung parenchymal loss is translated into systemic biological signals that reshape whole-body energy allocation. The degree of resection may determine the intensity of this shift, providing a mechanistic explanation for the procedure-dependent differences in long-term resilience observed in clinical trials 3.

## Discussion

Randomized trials in early stage non–small cell lung cancer (NSCLC) have fundamentally reshaped the surgical landscape. The JCOG0802/WJOG4607L trial demonstrated superior overall survival (OS) with segmentectomy compared to lobectomy, despite a higher incidence of local recurrence^3^. Crucially, this survival advantage was largely driven by a significant reduction in non-lung cancer-related deaths. This finding presents a biological paradox: if ventilatory capacity eventually recovers after lobectomy to a degree comparable to segmentectomy, why does the extent of resection dictate long-term non-lung cancer survival? We propose that the key to resolving this paradox lies not simply in the ventilation mechanism, but in the permanent loss of the pulmonary vascular bed and demonstrate one possible framework for explanation of the paradox.

As our framework suggests, anatomical resection induces a chronic “pulmonary hypertensive phenotype” characterized by sustained right ventricular (RV) afterload and endothelial shear stress. In established pulmonary arterial hypertension (PAH), such chronic cardiopulmonary strain is known to trigger systemic catabolic signaling, often involving GDF-15 as one potential mediator among several candidate interorgan pathways, such as angiotensin II, interleukin-6, or tumor necrosis alpha [45], which modify skeletal muscle metabolism. Although the application of GDF-15 as a stress marker in lung surgery still remains to be elucidated, we posit that a similar, albeit subclinical, “lung-muscle metabolic axis” is activated following major lung resection, creating a persistent systemic metabolic burden that accelerates fraility. In cases of right heart overload or right heart failure resulting from pulmonary circulatory overload, it has been found that a relative deficiency of natriuretic peptides such as ANP, BNP and CNP secreted from the heart promotes catabolic renewal of skeletal muscle [46] therefore it might be noted that the Lung-Muscle Axis is merely one stage of the metabolic network between organs [47, 48].

This perspective defines a dual-axis paradigm in surgical decision-making: oncological radicality versus systemic resilience of the patient. While extensive resection ensures local tumor control, it concomitantly exacerbates a “metabolic trade-off,” diverting energetic resources toward organ-level adaptation at the expense of somatic maintenance. Consequently, parenchyma-sparing surgery, such as segmentectomy, should be viewed not only as a means to preserve FEV₁ but also as a strategy to safeguard the pulmonary vascular reserve and, by extension, skeletal muscle integrity.

Understanding this axis opens new avenues for providing precision surgical care. The preoperative assessment of the pulmonary artery-to-aorta (PA/A) ratio, RV strain imaging, and baseline sarcopenia [49] indices could help identify patients at high risk for “metabolic phase-shifts” after resection. Targeted interventions, including cardiopulmonary rehabilitation and nutritional support, may help mitigate these systemic effects. Future research should prospectively integrate metabolic and muscle-centric endpoints into thoracic surgical trials to validate the lung-muscle axis. In the era of aging populations and increasing multimorbidity, radical yet parenchyma-sparing surgery must be established as a standard that ensures both oncological cure and long-term physiological homeostasis [50].

## Conclusion

The surgical invasiveness of lung cancer treatment must be re-evaluated beyond the traditional metrics of anatomical loss and acute perioperative stress. Our “lung-muscle metabolic axis” framework suggests that the extent of parenchymal resection dictates the magnitude of chronic physiological adaptation required to maintain systemic homeostasis. While ventilatory capacity may eventually normalize, irreversible reduction of the pulmonary vascular bed imposes sustained hemodynamic and metabolic strain. This persistent burden can trigger a systemic catabolic state in which skeletal muscle, acting as a critical metabolic reservoir, is progressively depleted, thereby increasing long-term vulnerability to non-cancer-related mortality.

Future surgical strategies should prioritize “metabolic preservation,” aiming to ensure an oncological cure while minimizing permanent disruption of the pulmonary vascular-metabolic axis. Integrating longitudinal monitoring of cardiopulmonary strain and muscle mass into clinical practice is essential to provide precision surgical care and improve the holistic prognosis of lung cancer survivors. Therefore, this framework should be regarded as a conceptual integrative model that warrants prospective clinical and mechanistic validation.

## References

[1] Graham EA, Singer J. Successful removal of a entire lung for carcinoma of bronchus. JAMA. 1933;101:1371–4.

[2] Cahan WG. Radical lobectomy. J Thorac Cardiovasc Surg. 1960;39:555–72. 13806783

[3] Saji H, Okada M, Tsuboi M, Nakajima R, Suzuki K, Aokage K, et al. JCOG0802/WJOG4607L randomized trial: segmentectomy vs lobectomy for small peripheral NSCLC. Lancet. 2022;399:1607–17. 35461558 10.1016/S0140-6736(21)02333-3

[4] Altorki N, Wang X, Kozono D, Watt C, Landrenau R, Wigke D, et al. Lobar or sublobar resection for peripheral stage IA non-small-cell lung cancer. N Engl J Med. 2023;388:489–98. 36780674 10.1056/NEJMoa2212083 PMC10036605

[5] Wang TW, Zhang Q, Cai Z, Xu Q, Lin J, Yeh H. Compensatory function change by segment-counting method in predicted postoperative pulmonary function at 1 year after surgery: systematic review and meta-analysis. BMJ Open Respir Res. 2024;11:e001855. 10.1136/bmjresp-2023-001855. 39622586 PMC11624756

[6] Shelley B, Glass A, Keast T, McErlane J, Hughes C, Lafferty B, et al. Perioperative cardiovascular pathophysiology in patients undergoing lung resection surgery: a narrative review. Br J Anaesth. 2023;130:e66–79. 35973839 10.1016/j.bja.2022.06.035 PMC9875905

[7] Ueda K, Tanaka T, Hayashi M, Li T-S, Tanaka N, Hamano K. Computed tomography-defined functional lung volume after segmentectomy versus lobectomy. Eur J Cardiothorac Surg. 2010;37:1433–7. 20153214 10.1016/j.ejcts.2010.01.002

[8] Suzuki H, Morimoto J, Mizobuchi T, Fujiwara T, Nagato K, Nakajima T, et al. Does segmentectomy really preserve the pulmonary function better than lobectomy for patients with early-stage lung cancer? Surg Today. 2017;47:463–9. 27484067 10.1007/s00595-016-1387-4

[9] Mizobuchi T, Chen F, Yoshino I, Iwata T, Yoshida S, Bando T, et al. Radiologic evaluation for volume and weight of remnant lung in living lung donors. J Thorac Cardiovasc Surg. 2013;146:1253–8. 23856207 10.1016/j.jtcvs.2013.05.038

[10] Wada H, Yoshida S, Suzuki H, Sakairi Y, Mizobuchi T, komura D, et al. Transplantation of alveolar type II cells stimulates lung regeneration during compensatory lung growth in adult rats. J Thorac Cardiovasc Surg. 2012;143:711–9. 22035964 10.1016/j.jtcvs.2011.09.024

[11] Ito T, Suzuki H, Wada H, Fujiwara T, Nakajima T, Iwata T, et al. Concordant pattern of radiologic, morphologic, and genomic changes in compensatory lung growth. J Surg Res. 2017;212:60–7. 28550923 10.1016/j.jss.2016.12.035

[12] Mammoto T, Muyleart M, Mammoto A. Endothelial YAP1 in regenerative lung growth through angiopoietin-Tie2 pathway. Am J Resp Cell Mol Biol. 2019;60:117–27. 10.1165/rcmb.2018-0105OC PMC6348720 30156429

[13] Eckle T, Kewley EM, Brodsky KS, Tak E, Boney S, Gobel M, et al. Identification of hypoxia-inducible factor HIF1A as transcriptional regulator of A2B adenosine receptor during acute lung injury. J Immunol. 2014;192:1249–56. 24391213 10.4049/jimmunol.1100593 PMC3946986

[14] Wei B, D’Amico T, Samad Z, Hasan R, Berry MF. The impact of pulmonary hypertension on morbidity and mortality following major lung resection. Eur J Cardiothorac Surg. 2014;45:1028–33. 24132298 10.1093/ejcts/ezt495 PMC4020383

[15] Deslauriers J, Ugalde P, Miro S, Ferland S, Bergerson S, Lacasse Y, et al. Adjustments in cardiorespiratory function after pneumonectomy: Results of the pneumonectomy project. J Thorac Cardiovasc Surg. 2011;141:7–15. 21168011 10.1016/j.jtcvs.2010.09.010

[16] Glass A, McCall PJ, Arthur A, Mangion K, Shelley BG. Pulmonary artery wave reflection and right ventricular function after lung resection. Br J Anaesth. 2023;130:e128–36. 36115714 10.1016/j.bja.2022.07.052 PMC9875909

[17] McCall PJ, Arthur A, Glass A, Jhonson M, Kinssella J, Shelley BG. The right ventricular response to lung resection. J Thorac Cardiovasc Surg. 2019;158:556–65. 30826095 10.1016/j.jtcvs.2019.01.067

[18] Nishikubo M, Kuroda S, Haga N, Nishioka Y, Shimizu N, Fukuda Y, et al. Pulmonary artery enlargement as a predictore of long-term prognosis in patients with resectred early-stage non-small cell lung cancer. JTCVS Open. 2024;23:266–75. 40061524 10.1016/j.xjon.2024.11.009 PMC11883713

[19] Nishikubo M, Tanaka Y, Tane S, Hokka D, Maniwa Y. Lobectomy increase postoperative pulmonary artery enlargement to a greater extent than segmentectomy. Ann Thorac Cardiovasc Surg. 2025;31:24–00083. 39880609 10.5761/atcs.oa.24-00083 PMC11781968

[20] Lewis GD, Pappagianopoulos PP, Systrom DM, Haley KJ, Camuso JM, Pappas AJ, et al. Impact of lung resection on pulmonary vascular reserve and exercise capacity. J Heart Lung Transpl. 2011;30:253–61.

[21] Rol N, Happé C, Beliën JAM, de Man FS, Westerhof N, Vonk-Noordegraaf A, et al. Vascular remodelling in the pulmonary circulation after major lung resection. Eur Respir J. 2017;50:1700806. 28860267 10.1183/13993003.00806-2017

[22] Okada M, Ota T, Okada M, Matsuda H, Okada K, Ishii N. Right ventricular dysfunction after major pulmonary resection. J Thorac Cardiovasc Surg. 1994;108:503–11. 8078343

[23] Harada H, Okada M, Sakamoto T, Matsuoka H, Tsubota N. Functional advantage after radical segmentectomy versus lobectomy for lung cancer. Ann Thorac Surg. 2005;80:2041–5. 16305841 10.1016/j.athoracsur.2005.06.010

[24] Tsuchiya A, Utsumoniya H, Kamigaichi A, Tsutani Y, Hamada A, Takeuchi M, et al. Determinatnts and mechanisms of postoperative effort intolerance in suspected lung cancer- An exercise-stress echocardiography study. Circulation J. 2026;10:1253. 10.1253/circj.CJ-25-0733 41708104

[25] Moonen JR, Chappell J, Shi M, Shinohara T, Li D, Mumbach MR, et al. KLF4 recruits SWI/SNF to increase chromatin accessibility and reprogram the endothelial enhancer landscape under laminar shear stress. Nat Commun. 2022;13:4941. 35999210 10.1038/s41467-022-32566-9 PMC9399231

[26] Evans CE, Cober ND, Dai Z, Stewart DJ, Zhao YY. Endothelial cells in the pathogenesis of pulmonary arterial hypertension. Eur Respir J. 2021;58:2003957. 33509961 10.1183/13993003.03957-2020 PMC8316496

[27] Wollert KC, Kempf T, Peter T, Olofsson S, James S, Johnston N et al. Prognostic value of growth-differentiation factor-15 in patients with non-ST-enevation acute coronary syndrome. Circulation 2007:115062–71. 10.1161/CIRCULATIONAHA.106.650846 17283261

[28] Prabhakar A, De Bie EM, Des Jardin JT, Ghatpande P, Graf S, Howard LS, et al. GDF15 is a putative biomarker for distinguishing pulmonary veno-occlusive disease and pulmonary hypertension. J Clin Invest. 2025;136:e199159. 41264364 10.1172/JCI199159 PMC12807463

[29] Garfield BE, Crosby A, Shao D, Yang P, Read C, Sawiak S, et al. Growth/differentiation factor 15 causes TGFβ-activated kinase 1-dependent muscle atrophy in pulmonary arterial hypertension. Thorax. 2019;74:164–76. 30554141 10.1136/thoraxjnl-2017-211440 PMC6467240

[30] Semmelmann A, Scheid S, Moneke I, Winroth D, Hell J, Burkle H, et al. Role of the perioperative growth differentiation factor-15 in identifying patients at high risk for postoperative pulmonary complications following thoracic surgery. J Cardiothorac Vasc Anesth. 2026;40:643–51. 41354610 10.1053/j.jvca.2025.10.021

[31] Desborough JP. Physiology of the stress response to surgery and trauma. Br J Anaesth. 2000;85:109–17. 10927999 10.1093/bja/85.1.109

[32] Moore FD, Ball MR. Metabolic response to surgery. Springfield III. Charls C Thomas; 1952.

[33] Isaka T, Ito H, Yokose T, Saito H, Narinatsu H, Adachi H, et al. Long-term changes in psoas muscle mass after lobectomy and segmentectomy for early-stage lung cancer. J Cachexia Sarcopenia Muscle. 2023;14:2540–9. 37740651 10.1002/jcsm.13328 PMC10751413

[34] Takamori S, Tagawa T, Toyokawa G, Shimokawa M, Kinoshita F, Kozuma Y, et al. Postoperative skeletal muscle loss and prognosis after lung cancer surgery. Ann Thorac Surg. 2020;109:914–20. 31655044 10.1016/j.athoracsur.2019.09.035

[35] Argiles JM, Campos N, Lopez-Pendrosa JM, Rueda R, Rodriguez-Manas L. Skeletal muscle regulates metabolism via interotgan crosstalk: Roles in health and disease. J Am Med Dir Assoc 2016;789096. 10.1016/j.jamda.2016.04.019 27324808

[36] Bodine SC, Latres E, Baumhueter S, Lai VK, Nunez L, Clarke BA, et al. Identification of ubiquitin ligases required for skeletal muscle atrophy. Science. 2001;294:1704–8. 11679633 10.1126/science.1065874

[37] Puthucheary ZA, Rawal J, McPhail M, Connolly B, Ratnayake G, Chan P, et al. Acute skeletal muscle wasting in critical illness. JAMA. 2013;310:1591–600. 24108501 10.1001/jama.2013.278481

[38] Choi MH, Yoon SB, Lee K, Song M, Lee IS, Lee MA, et al. Preoperative sarcopenia and post-operative accelerated muscle loss negatively impact survival after resection of pancreatic cancer. J Cachexia Sarcopenia Muscle. 2018;9:326–34. 29399990 10.1002/jcsm.12274 PMC5879976

[39] Argillander TE, Spek D, van der Zaag-Loonen HJ, van Raamt AF, van Duijvendijk P, van Munster BC. Association between postoperative muscle wasting and survival in older patients undergoing for non-metastatic colorectal cancer. J Geriatric Oncol. 2021;12:1052–8. 10.1016/j.jgo.2021.04.004 33858804

[40] Shen ZL, Liu Z, Zhang P, Chen WZ, Dong WX, Chen WH, Lin F, Zang WF, et al. Prognostic significance of postoperative loss of skeletal muscle in patients underwent coronary artery bypass grafting. Front Nutr. 2022. 10.3389/front2022.970729. 36118747 PMC9478409

[41] Harada T, Tatematsu N, Ueno J, Koishihara Y, Konishi N, Fukushima T, et al. Impact of early postoperative factors on changes in skeletal muscle mass after esophagectomy in older patients with esophageal cancer. Eur Geriatr Med. 2023;14:203–10. 36586085 10.1007/s41999-022-00735-0 PMC9902305

[42] Williams GR, Dunne RF, Giri S, Schachar SS, Caan B. Sarcopenia in the older adult with cancer. J Clin Oncol. 2021;39:2068–78. 34043430 10.1200/JCO.21.00102 PMC8260902

[43] Nagao A, Wakabayashi H, Maeda K, Kokura Y, Miyazaki S, Mori T et al. Respiratory sarcopenia and sarcopenic respiratory disability: concepts, diagnosis, and treatment. J Nutr Health Aging. 2021:507–15. 10.1007/s12603-021-1587-5 PMC7799157 33786569

[44] Damluji AA, Alfaraidhy M, AlHajiriN, Rohant NN, Kumar M, Malouf AC, et al. Sarcopenia and cardiovascular diseases. Circulation. 2023;147:1534–53. 37186680 10.1161/CIRCULATIONAHA.123.064071 PMC10180053

[45] Singh N, Kocana C, Schmidt E, Hepokoski M, Matthay MA, Stevens T. Mechanisms of lung crosstalk with End-Organs. Am J Physiol Lung Cell Mol Physiol. 2025;329:L419–27. 40839382 10.1152/ajplung.00175.2025 PMC12621758

[46] Gupta DK, Wang TJ. Natriuretic peptides and cardiovascular health. Circulation J. 2015;79:1647–55. 10.1253/circj.CJ-15-0589 PMC4893202 26103984

[47] Cui X, Liu H, Yu Z, Wang D, Wei W, et al. The role and mechanisms of myokines in sarcipenia: new intervention strategy for the challenges of aging. Fron Med. 2025;10:3389. 10.3389/fmed.2025.1665708 PMC12546142 41140691

[48] Argiles JM, Campos N, Lopez-Pedrosa JM, Rueda R, Rodriguez-Manas L. Skeletal muscle regulates metabolism via interorgan crosstalk: Roles in health and disease. J Am Med Dir Assoc. 2016;10:1016. 10.1016/j.jamda.2016.04.019 27324808

[49] Sun C, Hirata Y, Kawahara T, Kawashima M, Sato M, Nakajima J, et al. Diagnosis of Respiratory Sarcopenia for Stratifying Postoperative Risk in Non-Small Cell Lung Cancer. JAMA Surg. 2025;160:66–73. 39475952 10.1001/jamasurg.2024.4800 PMC11581747

[50] Wenner A-K, Andereggen L, Luedi NN. Molecular mechanisms diving precision medicine in perioperative care: Integrating inflammation, metabolism, and neuroimmunomodulation for personalized outcomes. Int J Mol Scie. 2025;26:12043. 10.3390/ijms262412043 PMC12733346 41465469

